# Case report: Characterizing of free-floating pigmented vitreous cyst using swept-source optical coherence tomography

**DOI:** 10.3389/fmed.2024.1428353

**Published:** 2024-08-30

**Authors:** Kexin Shi, Qichuan Yin, Yuxin Huang, Sifan Zheng, Yao Wang, Xingchao Shentu

**Affiliations:** ^1^The Eye Center, Second Affiliated Hospital of School of Medicine, Zhejiang University, Hangzhou, Zhejiang, China; ^2^Zhejiang Provincial Key Lab of Ophthalmology, Hangzhou, Zhejiang, China; ^3^GKT School of Medical Education, King’s College London, London, United Kingdom

**Keywords:** vitreous cyst, optical coherence tomography, B-scan ultrasound, slit lamp, fundus photography

## Abstract

**Aim:**

A free-floating vitreous cyst is a rare eye disease. This study aimed to find diagnostic imaging methods and imaging features for vitreous cysts.

**Methods:**

This article presents a case report along with a literature review of published cases of vitreous cysts. The case report describes a highly myopic 60-year-old woman with a pigmented, free-floating vitreous cyst in her right eye. A search of the PubMed database using the keywords “vitreous cyst” was performed to identify other cases reported in the literature and to summarize the imaging methods used to diagnose and visualize vitreous cysts and the imaging features of vitreous cysts.

**Results:**

A thorough ophthalmic examination was performed in the present case, including slit-lamp photography, B-scan ultrasound, broad line fundus imaging, spectral-domain optical coherence tomography (SD-OCT), and ultra-wide field SS-OCT. The literature review revealed the imaging methods used in previously reported cases of vitreous cysts in which ultra-wide field SS-OCT has the advantages of wide scanning depth and high imaging clarity.

**Conclusion:**

SS-OCT has an advantage over SD-OCT in providing intuitive morphological characteristic images for the diagnosis of posterior vitreous cysts. The comprehensive assessment of multimodal imaging examinations, including SS-OCT, is of significant value for the diagnosis and differential diagnosis of vitreous cysts.

## 1 Introduction

Vitreous cysts are rare ocular malformations. It can be either congenital or acquired. A vitreous cyst is usually detected during a routine ophthalmological examination or when it migrates to the visual axis and causes visual disturbances (1). Patients are often asymptomatic or may complain of intermittent blurred vision (2). In recent years, there have been only a few clinical case reports of vitreous cysts (3–10). The imaging studies in these reports are not uniform and lack systematic review.

Optical coherence tomography (OCT) is a non-invasive three-dimensional tomography technique that has been in use since 1991 (11). After continuous technological development, OCT has evolved from early time-domain OCT (TD-OCT) to spectral-domain OCT (SD-OCT) and finally to the latest swept-source OCT (SS-OCT) (12). OCT has evolved to enable detailed imaging of many intraocular structures (13). Wide field OCT (based on SS-OCT) has been used to detect non-perfused areas and retinal neovascularization in retinal vascular disorders (14); however, its application in vitreous cysts is rarely mentioned.

Here, we report a case of a vitreous cyst that has been observed and objectively assessed using multiple imaging methods, including a clear scan of the cyst contents using ultra-widefield SS-OCT. We also reviewed the relevant published literature on vitreous cysts and summarized the imaging methods that can objectively and qualitatively evaluate vitreous cysts. We concluded that SS-OCT can be used for the diagnosis of posterior vitreous cysts and has unique advantages.

## 2 Case presentation

A 60-year-old woman presented to the Eye Center, Second Affiliated Hospital of School of Medicine, Zhejiang University, complaining of blurred vision in both eyes for more than 3 years. She had a history of high myopia in both eyes for more than 30 years and had no previous history of ocular trauma, infectious eye disease, or abnormal tracing. Ophthalmological examination revealed an uncorrected visual acuity of 20/200 in the right eye and 20/100 in the left eye. The anterior chamber, iris, pupil, intraocular pressure, and eye movement were normal. The lenses of both eyes were opacified, and the fundus showed typical changes of high myopia. A pigmented cyst can be faintly seen in the right eye in the vitreous cavity, with a clear view obfuscated by cataract formation. The patient was followed up after microincision phacoemulsification combined with intraocular lens implantation in the right eye.

**Figure 1:**
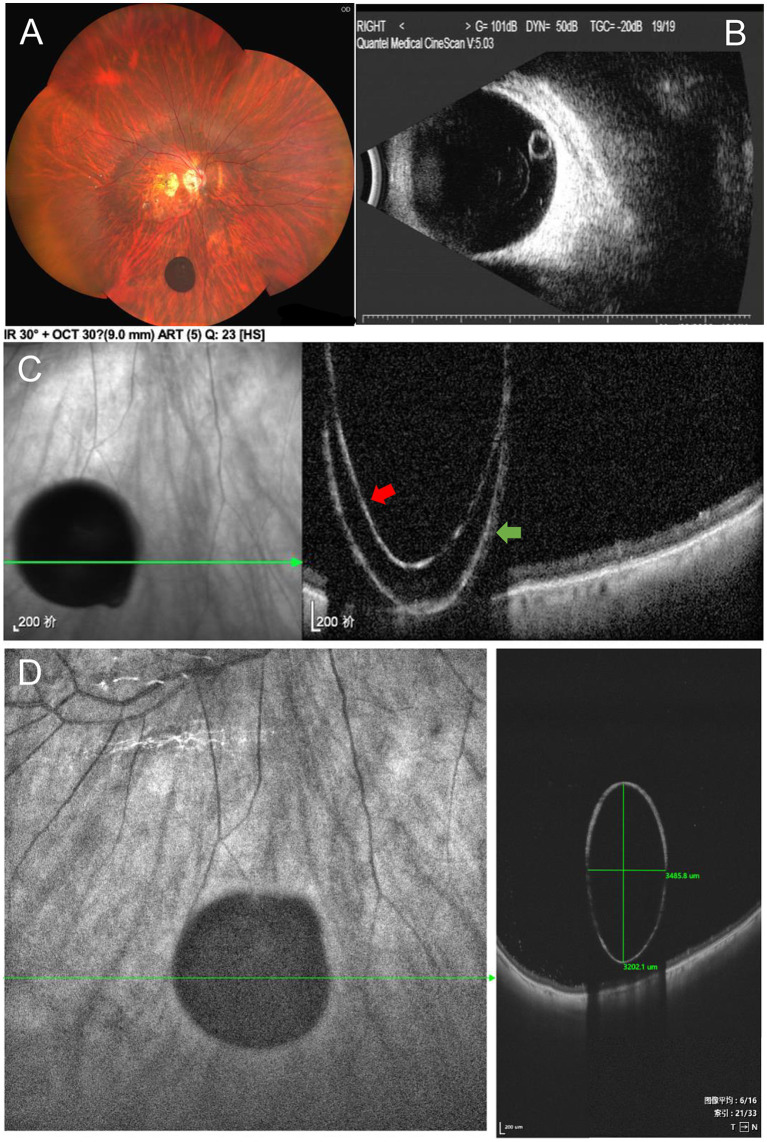
**(A)** Broad line fundus imaging showed a round, pigmented cyst. **(B)** Ultrasound B-scan showed the posterior-located vitreous cyst. **(C)** Spectral-domain OCT (SD-OCT) showed an image of a cyst with an inverse line. Red arrow: High-reflectivity band corresponds to the posterior edge of the vitreous cyst. Green arrow: The reflection image formed by exceeding the SD-OCT upper limit. **(D)** Ultra-wide field swept-source optical coherence tomography (SS-OCT), with 9 mm scan depth and 12 mm scan length, demonstrated a complete cyst image (3.5 mm × 3.2 mm in size) with a hyper-reflective enclosure, hypo-reflective content, and shadowing of the underlying retina.

We performed a series of imaging tests on the patient’s right eye after cataract surgery. Under the slit lamp, a free-floating cyst of approximately two optic disk diameters was observed in the vitreous body. The cyst was semi-transparent with a smooth surface and covered with brown pigment. When the patient rolled her right eye, the cyst moved with it (Supplementary Video 1). The broad line fundus imaging (CLARUS 500™; Carl Zeiss Meditec AG, Jena, Germany) showed a more clearly pigmented free-floating vitreous cyst with a defined boundary, spherical with small protrusions, located close to the mid-periphery retina (Figure 1A). We used an ultrasound B-scan to examine the cyst’s internal structure, which showed a cystic echo in the inferior vitreous cavity near the eyeball wall of the right eye, with an anechoic dark area inside. However, it was difficult to determine whether the cyst adhered to the eyeball wall (Figure 1B). SD-OCT (Figure 1C, Heidelberg Spectrails, Heidelberg, Germany) showed a hyper-reflective shell with hypo-reflective contents. Due to the depth limitation of SD-OCT scanning, some lesions were anterior to the upper limit of OCT and appeared as reflection images. The ultra-wide field SS-OCT (Figure 1D, VG200D; SVision Imaging, Henan, China), with a maximum scanning depth of up to 12 mm, demonstrated a complete oval-shaped, hyper-reflective thin shell filled with a homogeneous hypo-reflective liquid that did not adhere to the retina and shadowing of the underlying retina. The diagnosis was a pigmented vitreous cyst. As the patient had no visual impairment due to the cyst, she opted for conservative management. The cyst remained stable at the 3-month follow-up.

## 3 Discussion

Free-floating vitreous cysts were first described in 1899 (15), and only a few cases have been previously reported. Based on the limited reports, no gender difference was observed in patient profiles. Patients were primarily aged between 5 and 68, with most occurrences between 10 and 20 years (16). Vitreous cysts are primarily described as spherical or oval, rarely leaf-splitting, and in the 0.15 to 12 mm size range (17). Non-pigmented cysts have a yellowish appearance, while pigmented cysts are covered with sepia pigment particles. These vitreous cysts are usually asymptomatic and are often detected because of other ocular symptoms. However, when they involve the visual axis, they may cause blurred vision, shadows in the field of vision, or floating objects when moving (18). The embryonic origin of primary vitreous cysts remains controversial. Some researchers suggest that cysts may have originated from the primary hyaloid system, while others believe cysts originate in the iris, ciliary body, or retinal pigment epithelium (19, 20). Acquired vitreous cysts have been reported secondary to a variety of intraocular lesions. The most common reason is eye trauma (21), with other described causes including lattice degeneration, retinitis pigmentosa, retinal detachment, retinitis, choroidal disease, ciliary adenoma, ciliary ectopia, and intraocular infection (22–25).

Multimodal imaging studies are crucial for the diagnosis and differential diagnosis of vitreous cysts. Vitreous cysts in particular need to be differentiated from cysts caused by parasites, such as cysticercoid cysts (26). A slit-lamp examination can be used to observe the cyst’s shape, size, color, transparency, and activity (1). The cyst’s mobility can also be checked by having the patient turn her eye slightly. Fundus photography, especially broad line fundus photography, can qualitatively assess vitreous cysts. Adjacent fundus lesions can also be recorded (27), which may infer the etiology of vitreous cysts. However, the limitations of slit-lamp examination and fundus photography are that the internal components of the cyst cannot be detected, and the relationship between the cyst and the adjacent retina cannot be completely determined.

In addition, ultrasonography is also important for the diagnosis and measurement of the cyst. The typical B-ultrasound features of vitreous cysts describe the cysts as round or quasi-round, with moderate echo, thin wall, smooth echo wall, an anechoic dark area inside the cyst, and a positive posterior movement (28). These ultrasonographic features are distinguished from the dense circular echoes seen in the central portion of cysticercosis lesions in the vitreous cavity (29). However, the images of ultrasound are relatively rough and have low resolution, which cannot perfectly meet the needs of clinicians to explore the details of cysts.

In recent years, optical coherence tomography (OCT), as a non-invasive imaging technology, has been used in the diagnosis and treatment of vitreous cysts (30). OCT is a three-dimensional tomography technology that utilizes the biological tissue scattered light coherence principle for imaging and observation of living tissue. OCT has the advantages of non-contact, high resolution, and high speed (31). At present, the most commonly used is frequency-domain OCT. According to different methods of obtaining interference spectra, it is divided into spectral-domain OCT (SD-OCT), based on the spectrometer, and swept-frequency OCT (SS-OCT), based on a swept-frequency light source. OCT can display the internal structure of the vitreous cyst and the relationship between the cyst and the retina. Kevin et al. used SD-OCT for objective, qualitative assessment of large vitreous cysts. The OCT showed that the cysts cast shadows on both sides of the fovea, which was consistent with the annular scotoma symptoms described by the patient (32). Kevin et al. also suggested that OCT could be used to assess the risk–benefit ratio of vitrectomy in patients with symptomatic large vitreous floaters. Using SD-OCT, Dragnev et al. found that the cysts were multi-lobular and contained highly reflective material in addition to fluid on OCT scans (33). They speculated that these hyper-reflective spots correspond to premelonosomes, suggesting that the cysts originate from the primary hyaloid system.

Although SD-OCT is more frequently used in the evaluation of vitreous cysts, the shape of the cyst cannot be fully displayed due to the limited depth of SD-OCT scanning. The relationship between the cyst and surrounding tissues cannot be displayed simultaneously. Yonekawa et al. reported the use of SD-OCT to evaluate vitreous cysts, and the image showed that the anterior surface of the lesion was beyond the upper limit of SD-OCT, which appeared as a reflection image (34). However, SS-OCT uses a longer wavelength for imaging than SD-OCT, which has deeper penetration of biological tissue, and the swept-frequency light source has high instantaneous coherence, which can achieve a deeper longitudinal imaging range (35). Based on low sensitivity attenuation and larger longitudinal imaging range, SS-OCT can obtain a larger imaging depth than SD-OCT (36), which can facilitate imaging of the anterior segment, large-scale fundus imaging, and axial length imaging and feasibility. Using SS-OCT, Guo et al. reported a case of a vitreous cyst in a 48-year-old woman (37). The high-resolution images showed that the cyst was surrounded by a thin hyper-reflective wall and a posterior cortical anterior vitreous pocket. Unlike the typical liquid cyst interior morphology in our reported case, the cyst in their case was composed of a hyper-reflective septum with a slightly central agglutination. The hyper-reflective septum may be associated with premelonosomes. In addition, the ultra-wide field SS-OCT instrument used in our case offers super-depth imaging of up to 12 mm, which allows for a clearer display of the entire cyst, including its internal fluid structure and positional relationship with the retina, compared to the conventional SS-OCT instrument, thus enhancing clinical evaluation. Ultra-wide field SS-OCT can also obtain clear scan images at different levels, similar to pathological slices. Overall, SS-OCT meets the ever-increasing demands for detection speed, sensitivity, and functionality.

Some less commonly reported imaging methods can also be used in the diagnosis and differential diagnosis of vitreous cysts. Ocular ultrasound biomicroscopy (UBM) can be used to detect cysts arising from the iris/ciliary body (38) but is limited to the anterior segment. Infrared imaging has also been reported, which can show multiple areas of hyper-reflection on the cyst surface (39), but the image clarity is poor. Fluorescein angiography (FA) can be used to rule out overlying vascularization of the cyst (40), but only for cysts connected to the retina. Compared to other imaging methods, SS-OCT can display the external and internal structures of cysts across multiple layers, such as pathological slices, and provides a better differential diagnosis for the nature of cysts.

The treatment of vitreous cysts depends on the patient’s wishes, symptoms, degree of visual impairment, cyst characteristics, and location (41). Most vitreous cysts are asymptomatic or have a minimal visual impact, which only requires observation and regular follow-up. A minority with significant visual impairment or rapidly growing were considered for intervention. Treatment options include argon laser photocoagulation and neodymium:YAG (Nd:YAG) laser photocoagulation to remove the cyst (42, 43). Alternatively, vitrectomy surgery and pathological examination may also be performed to determine the benign or malignant nature of the cyst (44). However, these invasive treatments may be accompanied by serious complications, even with the relatively safe Nd:YAG laser, which has been reported to have caused iatrogenic cataract formation (45). Therefore, the treatment of vitreous cysts needs to be carried out with caution, and imaging plays a crucial role in guiding treatment decisions.

## 4 Conclusion

In conclusion, vitreous cysts are a rare condition that needs to be qualitatively examined and observed using the correct imaging methods, which are crucial for the diagnosis and treatment of the disease. We reported a case of a free-floating pigmented vitreous cyst, summarized the current clinical reports of imaging examination methods for vitreous cysts, and proposed the value of SS-OCT in the diagnosis of a posterior vitreous cyst.

## Data Availability

The original contributions presented in the study are included in the article/Supplementary material, further inquiries can be directed to the corresponding authors.
